# Analysis of Risk Factors for the Development of Gestational Diabetes Mellitus in a Group of Romanian Patients

**DOI:** 10.1155/2022/2367213

**Published:** 2022-06-02

**Authors:** Agnesa Preda, Adela Gabriela Stefan, Ionela Mihaela Vladu, Mircea-Catalin Fortofoiu, Diana Clenciu, Maria Fortofoiu, Ioan Ovidiu Gheorghe, Alexandru Cristian Comanescu, Maria Mota

**Affiliations:** ^1^University of Medicine and Pharmacy of Craiova, Romania; ^2^Clinical County Emergency Hospital of Craiova, Romania; ^3^Department of Diabetes Nutrition and Metabolic Diseases, Calafat Municipal Hospital, Calafat, Romania; ^4^Clinical Municipal Hospital “Philanthropy” of Craiova, Craiova, Romania; ^5^Public Health Department Gorj, Romania

## Abstract

**Introduction:**

Gestational diabetes mellitus (GDM) is caused by numerous risk factors, the most common being old age, obesity, family history of diabetes mellitus, GDM, history of fetal macrosomia, history of polycystic ovary syndrome or treatment with particular drugs, multiple births, and certain races. The study proposed to analyze the risk factors causing GDM.

**Method:**

In the study, we included 97 pregnant women to whom there was an OGTT performed between weeks 24th and 28th of pregnancy, divided into two groups, with GDM and without GDM. The statistical analysis was performed with SPSS 26.0, the tests being statistically significant if *p* value < 0.05.

**Results:**

The favoring risk factors for the onset of GDM were analyzed, with statistically significant differences between the GDM group and the group without GDM related to the delivery age (32.39 ± 4.66 years old vs. 28.61 ± 4.71 years old), history of fetal macrosomia (13.7% vs. 0%), presence of GDM during previous pregnancies (7.8% vs. 0%), HBP before pregnancy (9.8% vs. 0%), gestational HBP (17.6% vs. 0%), glycemia value at first medical visit (79.37 ± 9.34 mg/dl vs. 71.39 ± 9.16 mg/dl), and weight gain during pregnancy (14.61 ± 4.47 kg vs. 12.48 ± 5.87 kg).

**Conclusions:**

Identifying the risk factors for the GDM onset has a special importance, implying an early implementation of interventional measures in order to avoid the onset of GDM and associated maternal and fetal complications.

## 1. Introduction

Gestational diabetes mellitus (GDM) is defined by the American Diabetes Association (ADA) as “not previously known diabetes, diagnosed during the second or third trimester of pregnancy” [1]. The most common risk factors involved in the onset of GDM are represented by age over 40 years old, obesity, family history of diabetes mellitus (DM) in 1st degree relatives, history of GDM or fetal macorsomia, personal history of polycystic ovary syndrome or treatment with drugs like corticosteroids or antipsychotic drugs, multiple births, and race (Asian, African-American, Middle East, and some islands in the Pacific) [2]. An important role in the pathogenesis of GDM is played by insulin resistance and endothelial dysfunction, aggravated by unhealthy diet and sedentary lifestyle, which induce oxidative stress and the appearance of chronic inflammation and increasing inflammatory markers such as C-reactive protein, tumor necrosis factor-alpha (TNF-*α*), and interleukin (IL) 6. The recommendation to the pregnant woman, as early as possible of a vegetarian diet, rich in dietary fiber seems to decrease inflammation, oxidative stress, endothelial dysfunction, and insulin resistance. Mediterranean diet might favorably impact the onset of GDM and its complications, having a favorable role in metabolic control of pregnant women, decreasing the risk of maternal-fetal complications [3]. During the COVID-19 time period, more risk factors for GDM were added, such as prolonged stress, weight gain, as a result of movement and/or access to healthy food limitation, or even SARS-Cov-2 infection, which may lead to direct pancreatic lesions and insulin resistance, or it may even cause type 1 DM in predisposed women, through an immune mechanism. Starting a Mediterranean diet could limit the onset of GDM, by preventing gestational weight gain, immune system improvement, and modulation of IL-6, C-reactive protein, and nuclear factor (NF)-Kb [4]; the role played by diet and physical exercise in preventing GDM is also supported by Mijatovic-Vukas et al. [5]. COVID-19 pandemic led to changes in the diagnosis, supervision of the progression, and births in women with GDM, both through the limitation of medical care access and due to the pregnant woman self-limitation of contacts [6].

The purpose of the study was to analyze the risk factors favoring the onset of GDM in a group of Romanian patients.

## 2. Material and Method

### 2.1. Participants

We performed an epidemiological, prospective, noninterventional study, over a period of 2 and a half years (December 2018–April 2021); the study was conducted in Romania, Craiova city, including women monitored at two medical units: Emergency Clinical County Hospital and Clinical Municipal Hospital “Philanthropy”. We included in the study a group of 97 pregnant women monitored during pregnancy, in whom there was an oral glucose tolerance test (OGTT) performed with 75 g pulvis anhydrous glucose on 3 times, between weeks 24 and 28 of pregnancy. After the results of OGTT, the pregnant women were divided into 2 groups, namely, group 1: 51 pregnant women with GDM and group 2: 46 pregnant women without GDM.

The inclusion criteria were age over 18 years old, pregnant women who signed the informed consent for study inclusion and were monitored during pregnancy within the Emergency County Hospital of Craiova and the Clinical Municipal Hospital “Philanthropy.”

The exclusion criteria were represented by women with type 1 and 2 DM diagnosed before pregnancy, women who later gave birth outside the Clinical County Emergency Hospital of Craiova and the Clinical Municipal Hospital “Philanthropy,” of Craiova, women with severe comorbidities that may influence the maternal and perinatal outcome (kidney disease, neoplasia, anemia, thyroid disorders, etc.), and women who did not present to the follow-up visits after delivery.

All the pregnant women included in the study consciously signed an informed consent. The study was performed according to the ethical principles from the Helsinki Declaration—updated, according to the Good Clinical Practice (GCP), respecting the right to integrity, confidentiality, and giving the subject the option to withdraw from the study at any moment.

The data of every participant in the study included demographic characteristics, personal physiological history (number of pregnancies and previous deliveries, number of miscarriages, number of interrupted pregnancies, history of in utero fetal death or fetal macrosomia), and familial history. The pregnancies were considered interrupted if the fetus death occurred until the gestational age of 20 weeks. After this age, the fetus death was considered in utero fetal death.

The patients were physically examined, and there were anthropometric data recorded regarding weight and height; the body mass index (BMI) was calculated, previous to pregnancy, according to the following formula: BMI = weight (kg)/height^2^ (in meters). The gestational age was determined according to the echographic data and by calculating the duration from the first day of the last period. BMI was classified according to the guidelines of the World Health Organization (WHO) [7]. Blood pressure (BP) was measured by using an automatic sphygmomanometer in the subjects on a sitting position, after 10 minutes of rest. We considered the pregnant women having high blood pressure (HBP)in the study who presented systolic BP values ≥ 140 mmHg and/or diastolic BP values ≥ 90 mmHg and/or following a high blood pressure treatment at home. Gestational HBP was considered HBP diagnosed after 20 weeks of amenorrhea.

### 2.2. Blood Tests

The blood tests were represented by fasting plasma glucose (FPG) during first prenatal visit, subsequently followed by 3 measurements of a jeun, one hour and 2 hours glycemia after uploading 75 g anhydrous glucose within OGTT, performed between weeks 24 and 28 of pregnancy. FPGs were obtained after a fasting period of 8-12 hours. In women with GDM, there was an OGTT performed with 75 g anhydrous glucose, and there were determined a jeun and 2 hours glycemia, 4-12 weeks after delivery.

### 2.3. Evaluation of Gestational Diabetes

GDM diagnosis was established according to the International Association of Diabetes and Pregnancy Study Groups (IADPSG) (Table 1) [8].

In order to exclude a prediabetes or prior to pregnancy diabetes, we performed an a jeun glycemia during first prenatal visit, using the standard diagnosis criteria. 4-12 weeks after delivery, we performed an OGTT with 75 g glucose in all women with GDM, using the standard diagnosis criteria, outside pregnancy, in order to exclude a possible diabetes before pregnancy.

### 2.4. Statistical Analysis

The data were recorded on a computer, in a database, EXCEL, then transferred to Statistical Package for the Social Sciences (SPSS) 26.0 (SPSS Inc., Chicago, IL, USA), codified and analyzed using this program. All the data were analyzed according to the presence or absence of GDM in women included in the study.

The distribution of continuous variables were tested for normal values using the Kolmogorov-Smirnov test. Normal distribution data were presented as average ± standard deviation (SD); the data that did not have a normal distribution were presented as a median and interquartile range (IQR). In order to determine the statistical significance of the differences between the two groups, we used Student's *t* test for comparing the averages, respectively, and the Mann-Whitney *U* test for comparing the medians. The percentages between the two groups were compared by using the chi square test.

All the performed tests were considered statistically significant if they recorded a *p* value < 0.05.

## 3. Results

We analyzed the risk factors known in the literature as responsible for the GDM onset. For the studied groups, the characteristics related to heredocholateral and personal history are summarized in Tables 2 and 3.

Women with GDM had twice more frequently 1st degree relatives with type 2 DM than the ones without GDM, yet with no statistically significant differences (*p* = 0.073) (Table 2).

There was the analyzed physiological personal history of the pregnant women included in the study, a studied parameter being older age at delivery, when there were recorded high statistically significant differences between the groups, pregnant women with GDM being older than the ones who did not develop GDM (*p* < 0.001) (Table 2).

The statistical analysis of previous pregnancies did not identify statistically significant differences between the 2 groups, although women with GDM had a higher number of pregnancies (*p* = 0.169) (Table 2).

There were not recorded any statistically significant differences regarding the number of previous births (*p* = 0.228) (Table 2).

Regarding the number of previous miscarriages, there were more cases observed in the group with GDM, without any statistically significant differences between the two groups (*p* = 0.412) (Table 2).

The number of patients who presented interrupted pregnancies (until the age of 20 weeks of pregnancy) was higher in the group with GDM, still with no statistically significant differences between the 2 groups (*p* = 0.754) (Table 2).

In utero fetal death (after the age of 20 weeks of pregnancy) was found in a single pregnant woman with GDM, unlike the group without GDM, where there was no case, with a nonstatistically significant difference (*p* = 0.340) (Table 2).

Fetal macrosomia was found exclusively in the pregnant women with GDM, with a statistically significant difference (*p* = 0.009) (Table 2).

The pathological personal history was the next studied objective, the obtained results being described in Table 3.

Regarding obesity, we analyzed the BMI previous to pregnancy in women who developed GDM, in comparison to those who did not develop GDM, still with no statistically significant differences between the 2 groups (*p* = 0.734) (Table 3).

Also, we recorded the data regarding the presence of GDM in previous pregnancies, and we identified some differences at the limit of statistical significance (*p* = 0.05) in the group with GDM (Table 3).

HBP previous to pregnancy with high values detected during the first 20 weeks of amenorrhea was found exclusively in the group with GDM, a statistically significant difference (*p* = 0.029) (Table 3).

Even from the beginning of pregnancy and during its progression, there were a series of parameters synthesized in Table 4.

One of these parameters was the value of glycemia during the first prenatal visit. Its value in the pregnant women who developed GDM was higher than the one in the group of those who did not develop GDM, with a high statistically significant difference (*p* < 0.001) (Table 4).

During pregnancy, there was an excessive weight gain analyzed, and we observed statistically significant differences between the two groups, pregnant women who have developed GDM presenting a higher weight gain (*p* < 0.05) (Table 4).

Gestational HBP (diagnosed after 20 weeks of amenorrhea) was observed more frequently in pregnant women who developed GDM than in the ones without GDM, a statistically significant difference (*p* = 0.003) (Table 4).

Preeclampsia, as a pregnancy associated complication, was found only in the group with GDM, namely, in 58.3% of the patients (Table 4).

## 4. Discussions

Obesity is a risk factor commonly associated with the development of GDM [10, 11]. In our study, obesity was strictly found only in pregnant women with GDM, even though there were not recorded any statistically significant differences between the two groups;

Age at delivery time was highly correlated with a statistically significant risk for GDM, data which are in accordance with those in the literature [11, 12].

Similar to numerous studies, the family history of DM increases the risk for GDM development [13]. In our study, even though the number of pregnant women who developed GDM had a family history of DM in a higher percentage, there were not recorded any statistically significant differences.

History of fetal macrosomia, also known as a risk factor for GDM [14], was found in a higher percentage in pregnant women who developed GDM, similarly to the data in the literature.

Excessive weight gain during pregnancy is frequently quoted in the literature as a risk factor for the onset of GDM [11, 15]. In our study, there was a higher weight gain recorded in the case of pregnant women who developed GDM.

GDM was associated with the presence of gestational HBP, an important weight gain probably representing one of the connection factors, as there was no significant difference regarding the BMI prior to pregnancy.

Despite the fact that there were more pregnant women with GDM who presented a family history of type 2 DM, a higher number of previous pregnancies, births, and miscarriages, as well as a number of interrupted pregnancies, namely, in utero fetal death, in comparison to the pregnant women without GDM, still with no statistical significance, could explain the limitations of this study due to the low number of pregnant women included in the study. GDM represents a risk factor not only for a future development of type 2 DM, mainly, but also of early cardiovascular diseases; therefore, prevention measures are required [16]. More clinical studies showed the efficiency of inositols, mainly, myo-inositol, in the prevention and treatment of GDM. At present, inositols are considered candidates for classical insulin sensitizers, being useful in the prevention and treatment of GDM; they reduce insulin resistance, the need for insulin in GDM, also improving the lipidic profile [17–20].

## 5. Conclusions

In conclusion, we highlight the importance of identifying the risk factors for the GDM onset, early detection, and therapeutic intervention; the screening is required not only for pregnant women at risk but also of those out of risk, and the start of interventional measures as soon as possible, in order to prevent the onset of GDM and its associated complications.

## Figures and Tables

**Table 1:** Diagnosis criteria for GDM (OGTT with 75 g glucose).

	A jeun glycemia	1 hour glycemia (OGTT)	2 hours glycemia (OGTT)	Observations
GDM	≥92 mg/dl (5.1 mmol/l)	≥180 mg/dl (10 mmol/l)	≥153 mg/dl (8.5 mmol/l)	A single pathological value may support the GDM diagnosis

Reproduced from Medicina 2021, 57(11), 1170; doi:10.3390/medicina57111170. Analysis of maternal and neonatal complications in a group of patients with gestational diabetes mellitus [under the Creative Commons Attribution License/public domain] [9].

**Table 2:** Physiological heredocholateral and personal history.

		Without GDM	With GDM	*p*
Heredocholateral history of type 2 DM		8 (17.4%)	17 (33.3%)	0.073
Age at delivery time (years old)—*average* ± *D*S		28.61 ± 4.71	32.39 ± 4.66	**<0.001**

Age at delivery time (years old)	20-25	8 (17.4%)	1 (2%)	**<0.001**
	25-30	24 (52.2%)	14 (27.5%)	
	30-35	10 (21.7%)	16 (31.4%)	
	≥35	4 (8.7%)	20 (39.2%)	

No. of previous pregnancies	0 pregnancy	26 (56.5%)	20 (39.2%)	0.169
	1 pregnancy	14 (30.5%)	21 (41.2%)	
	2 pregnancies	6 (13%)	5 (9.8%)	
	3 pregnancies	0 (0%)	4 (7.8%)	
	≥4 pregnancies	0 (0%)	1 (2%)	

No. of previous deliveries	0 delivery	32 (69.6%)	29 (56.9%)	0.228
	1 delivery	14 (30.4%)	20 (39.2%)	
	2 deliveries	0 (0%)	2 (3.9%)	

No. of miscarriages	0 avorturi	42 (91.3%)	43 (84.3%)	0.412
	1 miscarriage	4 (8.7%)	5 (9.8%)	
	2 miscarriages	0 (0%)	2 (3.9%)	
	3 miscarriage	0 (0%)	1 (2%)	

No. of stopped pregnancies	0 pregnancy	40 (87%)	42 (82.4%)	0.754
	1 pregnancies	4 (8.7%)	6 (11.8%)	
	2 pregnancies	2 (4.3%)	2 (3.9%)	
	3 pregnancies	0 (0%)	1 (2%)	

History of in utero fetal death	Yes	0 (0%)	1 (2%)	0.340
History of fetal macrosomia	Yes	0 (0%)	7 (13.7%)	**0.009**

**Table 3:** Pathological personal history in the 2 studied groups.

		Without GDM	With GDM	*p*	Total
BMI (kg/m^2^)—*average* ± *SD*		22.75 ± 2.60	22.96 ± 3.44	0.734	22.86 ± 3.06

BMI (kg/m^2^)—categories	<18.5	6 (13%)	3 (5.9%)	0.075	9 (9.3%)
	18.5-25	31 (67.4%)	37 (72.5%)		68 (70%)
	25-30	9 (19.6%)	6 (11.8%)		15 (15.5%)
	≥30	0 (0%)	5 (9.8%)		5 (5.2%)

GDM in previous pregnancies	Yes	0 (0%)	4 (7.8%)	**0.05**	4 (4.1%)
	No	46 (100%)	47 (92.2%)		93 (95.9%)

HBP previous to pregnancy	Yes	0 (0%)	5 (9.8%)	**0.029**	5 (5.2%)
	No	46 (100%)	46 (90.2%)		92 (94.8%)

**Table 4:** Parameters observed during pregnancy.

		Without GDM	With GDM	*p*
Glycemia value at first prenatal visit (mg/dl)—*average* ± *SD*		71.39 ± 9.16	79.37 ± 9.34	**<0.001**

Glycemia value at first prenatal visit (mg/dl)—categories	<100	46 (100%)	51 (100%)	—
	100-125	0 (0%)	0 (0%)	
	≥126	0 (0%)	0 (0%)	

Weight gain (kg)—*average* ± *SD*		12.48 ± 5.87	5	**0.046**

Weight gain (kg)—categories	<10	8 (17.4%)	8 (15.7%)	0.449
	10-15	20 (43.5%)	16 (31.4%)	
	15-20	12 (26.1%)	21 (41.1%)	
	≥20	6 (13%)	6 (11.8%)	

Gestational HBP	Yes	0 (0%)	9 (17.6%)	**0.003**
	No	46 (100%)	42 (82.4%)	

Preeclampsia	Yes	—	7 (58.3%)	**—**
	No	—	5 (41.7%)	

## Data Availability

The data used to support the findings of this study are included within the article.
